# Does Administration of Low-Dose Aspirin Enhance the Efficacy of Psychotropic Drugs in Patients with Bipolar Disorder, Schizophrenia, and Schizoaffective Disorder?

**DOI:** 10.3390/ph19030435

**Published:** 2026-03-08

**Authors:** Lior Stern, Galila Agam, Rachel Shvartsur, Ali Alhoashla, Muhammad Abu Tailakh, Abed N. Azab

**Affiliations:** 1 Department of Nursing, School for Community Health Professions, Faculty of Health Sciences, Ben-Gurion University of the Negev, P.O. Box 653, Beer-Sheva 8410501, Israelabutaila@bgu.ac.il (M.A.T.); 2 Department of Clinical Biochemistry and Pharmacology, Faculty of Health Sciences, Ben-Gurion University of the Negev, P.O. Box 653, Beer-Sheva 8410501, Israel; galila@bgu.ac.il; 3 Department of Nursing, School of Health Sciences, Ashkelon Academic College, Yitshak Ben Zvi 12, Ashkelon 78211, Israel; shvartsurr@gmail.com; 4 Clalit Health Service, Southern District, Beer-Sheva 8410001, Israel; 5 Faculty of Health Sciences, Ben-Gurion University of the Negev, P.O. Box 653, Beer-Sheva 8410501, Israel; 6 Nursing Research Unit, Soroka University Medical Center, P.O. Box 151, Beer-Sheva 84101, Israel; 7 The School of Brain Sciences and Cognition, Ben-Gurion University of the Negev, P.O. Box 653, Beer-Sheva 8410501, Israel

**Keywords:** aspirin, bipolar disorder, brain, COX, inflammation, lithium, psychotic disorders, psychotropic medications

## Abstract

**Background/Objectives:** An extensive body of data suggests that inflammation may contribute to the pathophysiological mechanisms of psychiatric illness. Circumstantial evidence implied that low-dose aspirin (LDA) may enhance the therapeutic efficacy of psychotropic drugs. We examined whether LDA administration with psychotropic medications is associated with medication regimen stability and other therapeutic effects in patients with bipolar disorder (BD), schizophrenia, and schizoaffective disorder (SAD). **Methods:** This retrospective study analyzed data from Clalit Health Services’ Southern District database in Israel, including 1924 patients treated between 2017 and 2019. The Study Group consisted of patients treated with LDA plus psychotropic medications, whereas the Control Group included patients treated only with psychotropic medications. Study outcomes included suicide attempts and pharmacotherapy-related negative events, defined as psychotropic dose escalation, augmentation, or switching. **Results:** The study group included 137 patients (55% males, age 63.3 ± 12.3 years), and the control group included 1787 patients (60% males, age 47 ± 16.9 years). Significant differences were observed across nearly all outcomes, favoring the LDA co-treatment group. Patients in the study group exhibited lower rates of medication dosage increase (40 [29%] vs. 726 [40.5%], *p* = 0.01); fewer changes and/or additions of psychotropic medications (37 [26.9%] vs. 778 [43.5%], *p* < 0.001); and a non-significantly lower rate of suicide attempts (0 [0%] vs. 16 [0.9%], *p* = 0.53). **Conclusions:** Overall, LDA co-treatment was associated with better clinical outcomes among patients with BD, schizophrenia, and SAD. Follow-up large-scale epidemiological studies and prospective randomized clinical trials are needed to examine the therapeutic potential of add-on LDA to psychotropic medications.

## 1. Introduction

Mental illness affects millions of people globally, causing immense distress and placing a significant burden on both patients and their families [1,2]. Mental disorders are not only a source of personal suffering, but also result in substantial economic costs to individuals, families, and society at large [3]. The social and financial strain associated with mental health conditions emphasizes the need for comprehensive, long-term strategies to address this pervasive issue.

Bipolar disorder (BD) is a severe and chronic mental health condition that impacts 1–2% of the global population [4,5,6]. BD is characterized by alternating periods of acute mania and episodes of depression, which can range from a few days to several weeks in duration [5,7]. Additionally, BD is frequently associated with a higher incidence of comorbid medical conditions [8,9,10].

Schizophrenia, another chronic and debilitating psychiatric illness, affects approximately 1% of the population [2,11,12,13]. This disorder is marked by symptoms such as hallucinations, delusions, emotional flatness, and cognitive deficits [12,14]. A large-scale review, which examined data from 37 studies across 25 countries, found that schizophrenia not only severely impacts mental health but also detrimentally affects the physical health of those living with the disorder [15]. Similarly, schizoaffective disorder (SAD) is another debilitating mental illness, characterized by a combination of psychotic and mood-related symptoms [13,16,17].

Psychiatric disorders are typically managed using one or a combination of three treatment approaches: pharmacotherapy, psychotherapy, and electroconvulsive therapy [7,17,18,19,20,21,22,23,24,25,26,27,28]. Of these therapeutic modalities, pharmacotherapy remains a mainstay in the treatment of BD, schizophrenia, and SAD; it includes various psychotropic medications, such as antipsychotic drugs (e.g., chlorpromazine, haloperidol, perphenazine, clozapine, olanzapine and risperidone) [12,13,14,16,20,22,28,29,30], mood stabilizers (e.g., lithium, and the anticonvulsants valproate, carbamazepine and lamotrigine) [7,31,32,33,34,35,36], antidepressants (e.g., tricyclic antidepressants, monoamine oxidase inhibitors, selective serotonin reuptake inhibitors and serotonin-norepinephrine reuptake inhibitors) [37,38,39], among others [25,26,27]. Despite the availability of these treatments, a significant proportion of patients fail to respond adequately and/or experience numerous adverse effects, leading to poor treatment adherence and recurrence of symptoms [7,17,19,32,40,41,42,43,44,45,46,47]. These data underscore the need for new treatment strategies that are better tolerated and effective in a larger proportion of patients.

Various hypotheses have been proposed to explain the pathophysiology of BD, schizophrenia, and SAD. One prominent hypothesis suggests that inflammation plays a key role in the development of these disorders [48,49,50,51,52,53,54]. A growing body of evidence indicates that the inhibition of inflammation may enhance the therapeutic effects of mood stabilizers, antidepressants, and antipsychotic medications [55,56,57,58,59,60,61,62]. Support for the “inflammation hypothesis” of mental disorders comes from clinical trials that have shown that classic anti-inflammatory drugs, such as nonsteroidal anti-inflammatory drugs (NSAIDs) and corticosteroids, can have beneficial effects in patients with psychiatric disorders [60,63,64,65,66,67,68,69,70,71,72,73,74,75]. However, while the data supporting the role of inflammation in mental illness is promising, it remains inconclusive, as some studies have reported opposing findings [57,58,76,77].

Aspirin (acetylsalicylic acid), a widely used NSAID, is commonly employed to treat pain, fever, and inflammation [78]. Beyond its well-established cardiovascular benefits [79,80], low-dose aspirin (LDA) has also been found to offer other therapeutic effects. Long-term use of LDA has been associated with a reduced risk of various cancers [81,82,83,84,85,86,87]. It is widely accepted that LDA preferentially and irreversibly inhibits cyclooxygenase (COX)-1 [79]. Studies have shown that COX-1 plays a role in neuroinflammation-related conditions, and that even minimal inhibition of COX-1 by NSAIDs such as aspirin can contribute to their anti-inflammatory effects [88,89,90,91]. Additionally, long-term aspirin use has been suggested to weakly inhibit COX-2, further enhancing its anti-inflammatory properties [92]. Aspirin is also known to modulate inflammation through COX-independent mechanisms, such as inhibiting nuclear factor (NF)-κB [90,93,94]. In relation to mental illness, an early retrospective study found that aspirin was effective in reducing both positive and negative symptoms of schizophrenia [69]. Furthermore, indirect evidence suggested that combining LDA with lithium might improve lithium’s clinical efficacy among patients with BD [95].

Recent publications have also suggested that aspirin exhibits therapeutic effects among patients with mental disorders [96]. Nevertheless, it should be emphasized that some studies have reported contradictory results [97]. In this context, in two preclinical studies, we showed that LDA mitigated the typical renal side effects of standard-dose lithium [98] and augmented the anti-inflammatory effects of low-dose lithium while preserving lithium’s therapeutic behavioral outcomes [99]. Taken together, these findings suggest that add-on LDA may provide additional therapeutic benefits for adult patients with mental disorders.

The main objective of the present study was to examine the effects of LDA, co-administered with psychotropic medications, on parameters reflecting medication regimen stability—as a positive therapeutic indicator—in patients with BD, schizophrenia, and SAD.

## 2. Results

### 2.1. Basic and Clinical Characteristics of Study Participants

The basic sociodemographic characteristics and chronic morbidities of the study population are presented in Table 1. As seen, the mean age of the Study group was higher than that of the Control group. Moreover, patients in the Study group had a higher incidence of several chronic medical conditions (such as cerebrovascular accident, congestive heart failure, coronary artery disease, peripheral vascular disease, hypertension, renal disease, and diabetes mellitus) than those in the Control group. Importantly, as compared to the Control group, patients in the Study group had a significantly higher rate of BD.

### 2.2. Basic Laboratory Data of Study Participants

Table 2 presents the laboratory test values at baseline and six months later. As shown, the glucose level was higher in the Study group at both time points. In contrast, a lower level of magnesium was observed in the Study group compared to the Control group at both assessments, also the hemoglobin level was lower in the Study group at baseline.

### 2.3. Study Outcomes

Two major outcomes were evaluated: suicide attempts and pharmacotherapy-related negative events. As shown in Table 3, all medication regimen stability measures favored the Study group, indicating that patients who were co-treated with LDA had a better clinical status and experienced fewer negative psychiatric events. The rate of suicidal attempts was non-significantly lower in the Study group as compared to the Control group (0 [0%] vs. 16 [0.9%], *p* = 0.53; odds ratio [OR] = 0.39, 95% confidence interval [CI] = 0.02–6.54).

### 2.4. Subgroup Analysis

The major outcomes of the study were further examined according to psychiatric diagnosis (BD, schizophrenia, and SAD). As shown in Table 4, no suicide attempts were recorded in any diagnostic subgroup within the Study group. On the other hand, a low rate (~1%) of suicide attempts was recorded in the Control group, without a significant difference between the diagnostic groups. Rates of medication dosage escalations were similar across the three diagnoses, in the Study group as well as the Control group. On the other hand, in the Control group, there was a lower number of medication events “change and/or addition of another medication” in the BD group. This indicates that patients with BD in the Control group required fewer changes and/or additions to their medication regimen, suggesting a more stable clinical condition.

### 2.5. Age Stratification

To address the significant difference in baseline age between the Study group and the Control group, we performed an age-stratified analysis. The study population was divided into three clinically relevant strata: young adults (≤40 years), middle-aged (41–65 years), and older adults (≥66 years). As seen in Table 5, the association between LDA and the study outcomes did not differ substantially across age strata. Specifically, the direction and magnitude of the observed associations were similar across all age groups, suggesting that they were largely age-independent. In contrast, in the Control group, an age-related reduction in the rate of certain outcomes was observed. For instance, the rates of “change and/or addition of another psychiatric medication” and “amount of medication-associated negative events” were significantly lower among patients aged 66 years and older.

### 2.6. Correlation Analysis

Associations between LDA treatment and clinical outcomes were analyzed using Spearman’s correlation coefficients. Treatment with LDA showed a moderate negative correlation with the rate of medication dosage escalation (rho = −0.68, *p* < 0.05), a strong negative correlation with the addition and/or change in medication (rho = −0.91, *p* < 0.05), and a moderate negative correlation with the overall number of medication-associated negative events (rho = −0.49, *p* < 0.05). On the other hand, a weak positive correlation was found between LDA treatment and the occurrence of myocardial infarction (rho = 0.23, *p* < 0.05).

### 2.7. Multivariable Analysis for Identifying Predictors of the Study Outcomes

We performed a multivariable regression analysis to examine the influence of several selected independent sociodemographic and clinical variables on the outcomes of the study. Patients in the study group were divided into three subgroups according to the duration of LDA exposure: (i) six-weeks to six-months (n = 56), (ii) six-months to one-year (n = 53), and (iii) one-year to two-years (n = 28). Initially, a multivariable analysis was performed to test the impact of the selected variables on the outcome “medication dosage increase”. This analysis (presented in Table S1 in the Supplementary Data) revealed that six-weeks to six-months of LDA treatment, and having schizophrenia, were protective factors against the odds of increasing the dosage of psychiatric medication (OR: 0.309, 95% CI = 0.152–0.630, and, OR: 0.778, 95% CI = 0.619–0.978, respectively; Table S1). Contrastingly, Arab ethnicity was identified as a risk factor (OR: 1.435, CI = 1.022–2.014; Table S1). Similarly, a multivariable analysis was performed to examine the influence of the selected variables on the outcome “change and/or addition of another medication”. This analysis (presented in Table S2 in the Supplementary Data) revealed that older age, and, having BD, were significant protective factors against the occurrence of adding and/or changing the psychiatric medication (OR: 0.986, 95% CI = 0.98–0.992, and, OR: 0.733, 95% CI = 0.588–0.962, respectively; Table S2). On the other hand, having schizophrenia or SAD was found as a significant risk factor (OR: 1.364, CI = 1.039–1.791, and, OR: 1.551; CI = 1.14–2.11, respectively; Table S2).

The final model contained the occurrence of the sum of medication-associated negative events (i.e., medication dosage increase and change and/or addition of another medication) as a major outcome. As shown in Table 6, the model demonstrated several statistically significant protective factors against the occurrence of this outcome, including: older age, low socio-demographic status, having schizophrenia, having BD, and being treated with LDA between six-weeks to six-months. For instance, treatment with LDA for six-weeks to six-months was a significant protective factor against the occurrence of medication-associated negative events (OR: 0.426, 95% CI = 0.240–0.759; Table 6). Contrastingly, having SAD was found to be a risk factor for the occurrence of medication-associated negative events (Table 6).

## 3. Discussion

The primary objective of the present study was to evaluate the effects of LDA co-treatment with psychotropic medications on clinical parameters related to medication regimen stability among patients with BD, schizophrenia, and SAD. Our findings indicate that LDA treatment had a positive impact on nearly all tested measures.

Two major outcomes were evaluated: suicide attempts and pharmacotherapy-related negative events (Table 3). Co-treatment with LDA was associated with a lower incidence of pharmacotherapy-related negative events (including psychiatric medication dosage increase and change and/or addition of another psychiatric medication). Moreover, no suicide attempts were recorded in the LDA-treated group, as compared to 16 attempts in the Control group (a nonsignificant difference). These findings indicate that adjunctive LDA provides a notable benefit when added to standard pharmacotherapy for patients with BD, schizophrenia and SAD. Our findings align with previous research, which has shown that LDA usage correlates with a reduced occurrence of negative clinical events in patients with psychiatric disorders [60,95,100]. For example, Stolk et al. [95] reported that out of several anti-inflammatory drugs (including glucocorticoids and selective COX-2 inhibitors), only LDA co-treatment was associated with a reduction in medication-related negative events in patients with BD. Similarly, Kessing et al. [60] demonstrated that long-term LDA use among 321,350 patients with BD led to a significant decrease in hazard ratios for manic or bipolar episodes, with better results for treatment durations of 20 to 36 months.

In the present study, a higher incidence of BD was observed in the Study group as compared with the Control group (27% vs.16.1%, respectively; Table 1). In this context, patients in the Study group were older and had a higher incidence of several prominent medical comorbidities including cardiovascular disease, hypertension, chronic kidney disease (CKD) and diabetes mellitus (Table 1). The increased incidence of medical comorbidities may explain the need for LDA treatment in this group—most probably for secondary cardiovascular prevention (~22% of the patients in this group had a previous myocardial infarction, as compared to only 2% in the Control group; Table 1). Thus, our finding that there were more BD patients in the Study group is not surprising and is consistent with previous studies suggesting a bidirectional association between medical illnesses (such as cardiovascular disease and diabetes mellitus) and BD [101,102,103,104,105,106,107,108]. Interestingly, a subgroup analysis according to psychiatric diagnosis revealed that BD patients in the Control group had a lower incidence of “change and/or addition of another medication” events (Table 4), suggesting a more stable clinical condition. This result may be explained, at least in part, by the younger age of the patients in this subgroup and their lower incidence of medical comorbidities, which may indicate a less severe form of mental illness.

Furthermore, a previous preclinical study conducted in our laboratory demonstrated that LDA mitigated the typical nephrotoxic effects of lithium [98]. Thus, we hypothesized that patients treated with LDA would have lower creatinine levels, indicating better renal function. However, it was found that the levels of creatinine did not significantly differ between the groups; actually, they were non-significantly higher among patients of the Study group. The slightly higher incidence of CKD among LDA-treated patients (Table 1)—likely related to their older age, greater burden of comorbidities, and possibly a higher rate of lithium treatment due to the higher prevalence of BD in this group—may have contributed to the lack of an association between LDA treatment and reduced creatinine levels in the present study. Another seemingly intriguing result of the present study was a weak positive correlation between LDA treatment and the occurrence of myocardial infarction (r = 0.23, *p* < 0.05). Nonetheless, it should be emphasized that patients treated with LDA are inherently at higher risk for major adverse cardiovascular events (MACEs), which is why LDA was prescribed as a preventive measure.

To account for the substantial baseline age difference between the Study group and the Control group (mean age ± SD: 63.25 ± 12.26 vs. 47.01 ± 16.87, respectively), we performed an age-stratified analysis. This stratification allowed us to evaluate the association between LDA and medication-associated negative events within homogenous age groups, thereby minimizing age-related confounding. The analysis clearly revealed that the protective effects of LDA are not age-related, as there were no significant differences between the LDA-treated age groups (Table 5). On the other hand, in the Control group, some of the tested outcomes were significantly associated with age, with lower rates observed among patients aged ≥66 years (Table 5). Moreover, as discussed below, these findings are consistent with the multivariable analysis, which indicated that older age was associated with a reduced risk of medication-associated negative events. Thus, the age-stratified analysis suggests that the observed associations between LDA and study outcomes are generally consistent across age groups and are not merely a reflection of the age disparity between the treatment and control cohorts.

A multivariable regression analysis was performed to evaluate the impact of selected sociodemographic and clinical variables on the outcomes of the study. The final model contained the occurrence of the combined outcome: “medication-associated negative events”. The model showed that there were several protective factors against the occurrence of this outcome, including: older age, low socio-demographic status, being treated with LDA between six weeks to six months, having schizophrenia, and having BD (Table 6). The finding that older age is a protective factor influencing medication regimen stability is surprising, given the age-related increase in the incidence of psychiatric disorders, inflammatory diseases/neuroinflammation, and other comorbidities [109,110,111]. However, this finding is consistent with previous studies demonstrating an inverse association between age and symptom severity in psychiatric disorders [112,113,114]. For example, a multicenter longitudinal study in patients with schizophrenia identified younger age as a negative factor and a predictor of relapses [112]. In this context, Peters et al. [113] suggested that a decline in borderline traits across adulthood may explain the reduced symptom severity observed in patients with schizophrenia. Similarly, Goldberg et al. [114] found that the lower severity of depressive symptoms observed in older adults was largely driven by reduced cognitive-affective symptoms in this age group. On the other hand, our observation that low socioeconomic status was associated with a lower incidence of negative events contradicts most of the existing literature [115,116,117]. For example, a systematic review on antipsychotic medication adherence in BD and schizophrenia patients identified low socioeconomic status as a significant risk factor for nonadherence, which is usually associated with worse clinical outcomes [116]. The discrepancy between our findings and those of previous studies may be attributable to the study design, which included only patients with high medication adherence and excluded those with low adherence [118]. The result regarding BD is consistent with previous findings, suggesting that, in general, patients with BD tend to have a more stable clinical course and better social and functional outcomes than patients with schizophrenia or SAD [119,120,121,122]. Consistently, our multivariable regression analysis showed that SAD was a risk factor for the occurrence of medication-associated negative events (Table 6).

An important finding of the multivariable regression analysis was that treatment with LDA for six-weeks to six-months was a significant protective factor against the occurrence of medication-associated negative events (OR: 0.426, 95% CI = 0.240–0.759, *p* = 0.004; Table 6). However, the analysis indicated that the protective effect of LDA was not duration-dependent, as treatment durations of six-months to one-year and one to two years were not associated with a significant protective effect. This result is somewhat surprising; however, particularly for antiplatelet drugs such as aspirin (and others, including clopidogrel, prasugrel, and ticagrelor), there is evidence indicating that their therapeutic benefit is not necessarily duration-dependent in the sense that longer treatment duration leads to better outcomes [123,124,125,126]. Accumulating evidence suggests that shorter treatment durations are associated with better clinical outcomes, including a reduction in MACEs and a lower incidence of bleeding events [123,124,125,126]. Whether this principle also applies to the prevention of negative psychiatric events among LDA-treated patients with mental illness is currently unknown and remains to be determined.

Another interesting finding of the present study is the possible association between LDA use and the incidence of suicide attempts—no suicide attempts were recorded in the LDA-treated group, compared to 16 attempts in the Control group (Table 3). However, it is important to emphasize that the difference between the groups was not statistically significant (*p* = 0.53; OR = 0.39, 95% CI = 0.02–6.54). While this result may be of clinical interest, the study is underpowered and exploratory in nature, and no causal conclusions can be drawn in this regard. Additionally, given the unavailability of data on suicide deaths, caution is warranted in drawing definitive conclusions regarding the relationship between LDA use and suicidality. A suicide attempt is a serious event among patients with mental illness, as it represents an eminent risk factor for death by completed suicide [127,128]. Thus, even though the difference between the groups was not statistically significant, preventing even a single suicide could have a profound impact on patients’ families and communities. If future studies confirm a potential association between add-on LDA and reduced suicidal behavior, this could have important clinical implications for suicide prevention among these patients.

The antiplatelet effects of LDA have long been recognized in the secondary prevention of thrombotic events, particularly among patients with cardiovascular disease [78]. It is reasonable to assume that all patients in our study who received LDA did so for their cardiovascular conditions, rather than for psychiatric indications. Therefore, any observed psychiatric benefits of LDA should be considered a supplementary advantage, in addition to its well-established cardiovascular protective effects. While the results of this study, together with previous findings [60,95,96], suggest potential benefits, the key question remains: should LDA be prescribed to psychiatric patients even in the absence of cardiovascular indications? Based on our findings, as well as those of others, the answer seems to be affirmative. Nevertheless, addressing this question definitively will require future studies that are rigorously designed as randomized, double-blind, placebo-controlled trials. Additionally, follow-up epidemiological studies are needed, using a database with larger cohorts. As noted above, a recent double-blind, placebo-controlled randomized trial in patients with BD found that add-on LDA did not provide any benefit [97]. However, the negative results of this study may be explained, at least in part, by two factors: First, it enrolled a relatively medically healthy population—young, newly diagnosed BD patients with few comorbidities, particularly cardiovascular disease—contrasting with the LDA-treated group in the present study, which included older patients with a high incidence of comorbidities, including cardiovascular (Table 1). Second, it used a relatively “high-low dose” of aspirin (150 mg/day), which may reduce its preferential inhibition of COX-1 and thereby diminish its unique pharmacological advantage [79,129]; this also differs from the most commonly prescribed dose of LDA in Israel, which is 100 mg/day. Third, the study by Bruun et al. [97] included only patients with BD, whereas our study also included patients with schizophrenia and SAD; in this regard, it is noteworthy that our subgroup analysis (Table 4) appears to indicate that BD patients were the least likely to benefit from co-treatment with LDA.

**Figure 1:**
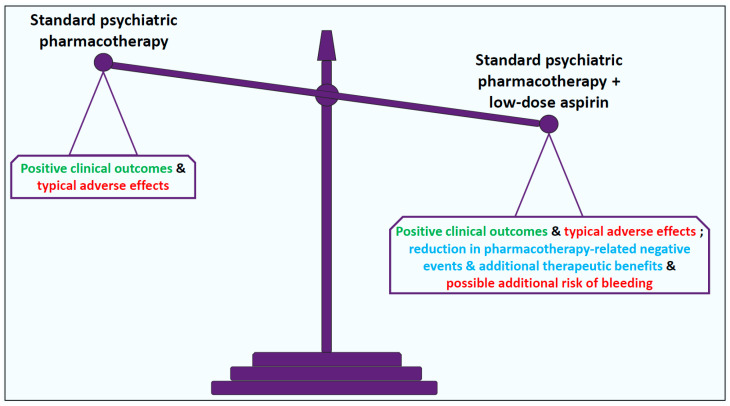
Illustrative demonstration of the additional benefits of adjunctive LDA in psychiatric patients receiving psychotropic medications. The addition of LDA to psychiatric medications reduces the risk of medication-associated negative events (present study) and may have additional therapeutic benefits, such as the mitigation of medication-related side effects [98], attenuation of major adverse cardiovascular events [79,80], and a reduction in the incidence of various cancers [81,82,83,84,85,86,87,135,136], among others [130,131,132,133,134]. However, long-term use of LDA may increase the risk of bleeding events [79,80,123,124,125,126]. Abbreviation: LDA, low-dose aspirin.

Given the known additional therapeutic benefits of LDA, including its potential to reduce cancer incidence [81,82,83,84,85,86,87] and its beneficial effects on various obstetric and gynecological conditions [130,131,132,133,134], this may represent another reason to consider prescribing LDA to adult psychiatric patients (see Figure 1 for illustration). Interestingly, recent data suggest that LDA exerts potent anti-metastatic effects in patients with cancer [135,136]. It has been shown that platelet-derived thromboxane A_2_ suppresses the ability of T cells to eliminate cancer metastases, thereby promoting metastatic progression; in contrast, LDA treatment reversed this effect, resulting in enhanced anti-metastatic and anticancer activity [135]. As to the potential mechanisms underlying the therapeutic benefits of LDA in psychiatric patients, and given the strong association between inflammation and mental illness, it is reasonable to assume that the anti-inflammatory properties of aspirin—even at low doses—may contribute to its therapeutic efficacy [69,137,138,139]. Moreover, aspirin treatment has been associated with neuroprotective effects and mitigation of oxidative stress and its related cellular damage [69,137,140,141].

The present study has several limitations inherent to its retrospective design. First, while we employed a robust multivariable logistic regression model to adjust for a wide range of potential confounders, including age, gender, socio-demographic factors, and specific somatic and psychiatric comorbidities, the possibility of residual confounding cannot be ruled out. Specifically, owing to the nature of the database, we lacked granular data on healthcare utilization frequency and the precise intensity of baseline psychotropic regimens, both of which could influence the observed associations. Nevertheless, by adjusting for socio-demographic factors and the primary psychiatric diagnosis, we aimed to approximate healthcare access and baseline illness severity. Second, beyond differences in baseline characteristics, there was also an imbalance in group size. In this regard, although propensity score methods were considered to address baseline imbalances, we retained our primary multivariable approach to preserve the full sample size and provide a clear, independent assessment of the effect of LDA treatment duration. This approach was complemented by the age-matched stratification that was performed. Third, although psychiatric hospitalization is an important clinical outcome, it was not documented in the medical records available to us; therefore, we were unable to examine it as a study outcome. Similarly, data on suicide deaths were not available. Suicide is a major adverse clinical outcome among patients with psychiatric disorders; therefore, the inability to assess the impact of LDA treatment on suicide mortality represents an important limitation of the present study. Fourth, although we excluded subjects with documented chronic use of nonsteroidal anti-inflammatory drugs, it is possible that some patients regularly used these medications through over-the-counter routes, which may have biased the study results. Nevertheless, it is reasonable to assume that such use occurred in both study groups; therefore, its overall impact on the findings is likely marginal or negligible.

## 4. Materials and Methods

### 4.1. Study Design, Population, and Data Sources

We conducted a retrospective study including patients with BD, schizophrenia, or SAD, who were treated at Clalit Health Services (CHS)—Southern District, Israel, and met the inclusion criteria of the study. CHS is the largest health maintenance organization in Israel, serving nearly 4.6 million enrollees. Data were obtained from the CHS database for the years 2017–2019 (consecutive sampling). The dataset was used to form two groups: (1) Study group—patients treated with a combination of LDA and psychotropic medication(s), and (2) Control group—patients treated only with psychiatric medication(s), without LDA. For the purposes of the present study, LDA was defined as a daily dose of 75–150 mg aspirin in adult patients, consistent with previous studies [79,80]. In this regard, in Israel, the vast majority of patients treated with LDA for secondary prevention of adverse cardiovascular events are prescribed 100 mg per day.

*Inclusion criteria*—Patients 18 years of age or older with one of the following diagnoses: BD, schizophrenia, or SAD, and were treated simultaneously for at least six weeks with one or more of the following psychiatric medications: lithium, valproate, carbamazepine, lamotrigine, chlorpromazine, haloperidol, perphenazine, thioridazine, fluphenazine, clotiapine, zuclopenthixol, clozapine, olanzapine, risperidone, aripiprazole, lurasidone, quetiapine, ziprasidone, amisulpride—with or without LDA.

*Exclusion Criteria*—(1) Patients with chronic inflammatory or immune diseases such as rheumatoid arthritis, systemic lupus erythematosus, inflammatory bowel disease, psoriasis, among other; (2) patients with autoimmune diseases; (3) chronic use (over a week) of medications that influence the immune system or possess anti-inflammatory effects (except LDA) during the study period; (4) psychiatric comorbidity—patients who had two or more psychiatric diagnoses; or (5) patients with immune deficiency due to conditions such as human immune-deficiency virus, immune suppressive treatment, etc.

*Exposure to LDA*—The effectiveness of psychiatric medication(s) treatment is usually decided 4–6 weeks after commencement of treatment [142,143]. Based on this, we established a cut-off of six weeks of LDA treatment as an inclusion criterion for the Study group. In this context, patients entered the study on a rolling basis during 2017–2019. For the Study group, the index date was defined as the date on which each patient completed six consecutive weeks of LDA treatment. To confirm this exposure, a six-week pre-index period was examined for each patient; for early entrants, this window could extend into late 2016. Events occurring during this pre-index period were *not* counted in the final analysis and did not affect eligibility. Control patients were assigned corresponding index dates and pre-index windows. Follow-up for outcomes began at each patient’s index date, ensuring alignment of follow-up between groups and minimizing immortal time bias. Thus, we stratified for the intensity of exposure to LDA according to the duration of treatment. Patients in the Study group were divided to three sub-groups according to the duration of LDA exposure: 1–6 months, 7–12 months, and 13–24 months. On the other hand, we did not stratify by LDA dose, as several studies [79,80] have shown no significant difference between the various aspirin doses within the 75–150 mg per day range. Information regarding the purchase of psychiatric medications and aspirin (as a measure of patients’ adherence to treatment) was obtained from the CHS database using Clalit’s MDClone-powered platform (https://www.mdclone.com), using the Anatomical Therapeutic Chemical (ATC) Classification.

### 4.2. Enrollment to the Study

**Figure 2:**
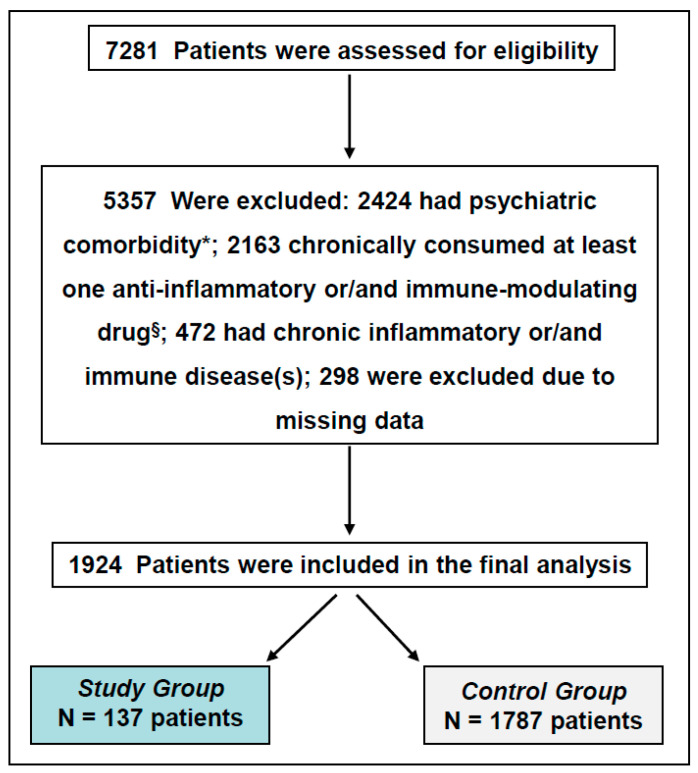
Flowchart of enrollment in the study. Eligible subjects were treated with at least one of the following psychiatric medications: Lithium, carbamazepine, valproic acid, lamotrigine, chlorpromazine, haloperidol, perphenazine, thioridazine, fluphenazine, zuclopenthixol, clozapine, olanzapine, risperidone, aripiprazole, quetiapine, ziprasidone, amisulpride, clotiapine, lurasidone. * Patients who had at least two psychiatric diagnoses; ^§^ chronic use for ≥1 week of at least one anti-inflammatory and/or immune-modulating medication (see text for more details).

A total of 7281 subjects who had one of the studied psychiatric diagnoses (BD, schizophrenia, or SAD) and were treated with a psychotropic medication during the years 2017–2019 were assessed for eligibility. Figure 2 presents the flowchart of the study enrollment process. A total of 1924 patients were included in the final analysis, comprising 137 in the Study group and 1787 in the Control group.

### 4.3. Study Outcome Measures

The present study evaluated two major outcomes indicative of clinical deterioration and/or treatment non-responsiveness: suicide attempts and pharmacotherapy-related negative events. These outcomes were compared between patients receiving combined treatment with LDA and psychiatric medications, and those treated with psychiatric medications alone. Of note, “psychiatric hospitalization” was originally included as an additional study outcome. However, initial analysis demonstrated substantial inconsistency in its documentation, precluding its reliable assessment; therefore, it was not included in the final analysis. The following parameters were recognized as pharmacotherapy-related negative events: (i) psychiatric medication(s) dosage increase, (ii) addition of other psychiatric medication(s); and (iii) switching to another psychiatric medication(s). We divided the outcome “medication dosage increase” into three categories: The first included patients who did not have any change in the psychiatric treatment; the second included patients with one or two dosage increase/s; and the third, included patients with three and more dosage increases. This division was based on the assumption that the more the dosage increases needed, the more likely his/her disease condition is unstable/difficult. Similarly, the outcomes “addition and/or change in psychiatric medication(s)” were also divided into three categories: (1) Patients who did not have an addition or change in psychiatric medication(s); (2) patients with one to three addition/s and/or change/s; and (3) patients with four or more additions and/or changes. This cutoff was determined based on the distribution of the data obtained.

### 4.4. Statistical Analysis

Continuous variables (e.g., age) were analyzed using Student’s t-test, and categorical variables (e.g., sociodemographic characteristics, medication(s) use, and complications) were analyzed using the Chi-square test. Categorical variables are presented as numbers and percentages; continuous variables as mean ± standard deviation (SD) or median (IQR) if abnormally distributed Spearman’s correlation was used to measure the strength and direction of associations between the tested variables.

*Sub-group analysis*: We examined the outcomes of the study according to the three sub-groups of patients—BD, schizophrenia, and SAD.

*Age stratification:* The study population was divided into three clinically relevant strata: young adults (≤40 years), middle-aged (41–65 years), and older adults (≥66 years). This stratification allowed us to evaluate the association between LDA and medication-associated negative events within homogenous age groups, thereby minimizing age-related confounding. For each stratum, we compared the frequency of the following parameters: suicide attempts, medication dosage increases, change and/or addition of another psychiatric medication(s), and amount of medication-associated negative events.

*Multivariate analysis:* We examined each type of negative psychiatric event—suicide attempts and pharmacotherapy-related negative events—as a dichotomous variable (for example, medication dosage increase: yes = 1; no = 0) and, also, combined the outcomes as a dichotomous variable (negative psychiatric events: yes = 1; no = 0). To examine selected predictors according to the results of the univariate analysis and according to what was regarded as clinically important, we used a logistic regression model. OR and 95% CI were calculated for outcomes. All statistical analyses were performed using R Statistical Software (version 4.2.2; R Core Team, 2022). In all analyses, a *p*-value < 0.05 was considered statistically significant.

## 5. Conclusions

In summary, the present study found that LDA co-treatment with psychotropic medications was associated with more favorable outcomes across almost all tested indicators among patients with BD, schizophrenia, and SAD. However, given the retrospective observational design of the study, these findings should be interpreted with caution as the observed associations may be influenced by selection bias and residual confounding. Future prospective, randomized, double-blind, placebo-controlled clinical trials are needed to assess the safety and efficacy of the combined treatment in patients with mental illness.

## Figures and Tables

**Table 1:** Demographic characteristics and chronic diseases of the study population.

Characteristic	Study Group n = 137	Control Group n = 1787	*p*-Value
Age (years), mean ± SD	63.25 ± 12.26	47.01 ± 16.87	<0.001
Gender, number (%) *			0.265
Female	62 (45.3)	716 (40)	-
Male	75 (54.7)	1072 (60)	-
Ethnicity, number (%)			0.362
Jews	119 (86.9)	1548 (86.6)	-
Arabs	12 (8.8)	193 (10.8)	-
Other	6 (4.4)	46 (2.6)	-
Socioeconomic status, number (%)			0.682
Low income	30 (21.9)	442 (24.7)	-
Medium income	92 (67.2)	1117 (62.5)	-
High income	9 (6.6)	154 (8.6)	-
Chronic Disease, number (%)			
Cerebrovascular accident	49 (35.8)	148 (8.3)	<0.001
Congestive heart failure	19 (13.9)	39 (2.2)	<0.001
Peripheral vascular disease	13 (9.5)	38 (2.1)	<0.001
Old Myocardial infarction	30 (21.9)	39 (2.2)	<0.001
Coronary artery bypass grafting	3 (2.2)	6 (0.3)	0.016
Angina pectoris	29 (21.2)	57 (3.2)	<0.001
Hypertension	99 (72.3)	415 (23.2)	<0.001
Renal disease	31 (22.6)	125 (7)	<0.001
Chronic pulmonary disease	54 (39.4)	449 (25.1)	<0.001
Liver disease	0 (0)	3 (0.2)	1
Diabetes mellitus	81 (59.1)	383 (21.4)	<0.001
Psychiatric Disease, number (%)			
Schizophrenia	75 (54.7)	1073 (60)	0.259
Bipolar disorder	37 (27)	288 (16.1)	0.002
Schizoaffective disorder	25 (18.2)	426 (23.8)	0.166

* Data are presented as number (%) or mean ± STD.

**Table 2:** Laboratory values.

	Start of Follow-Up			At Six Months of Follow-Up		
Characteristic	Study Group (n = 137) *	Control Group (n = 1787) *	*p*-Value	Study Group (n = 137) *	Control Group (n = 1787) *	*p*-Value
WBC	7.67 ± 2.24	7.73 ± 2.53	0.81	8.14 ± 2.86	7.97 ± 2.84	0.64
HB	13.09 ± 1.86	13.53 ± 1.68	0.01	13.08 ± 2.05	13.32 ± 1.81	0.32
PLT	236.74 ± 76.93	245.15 ± 75.36	0.28	233.36 ± 79.6	238.29 ± 79.42	0.64
Creatinine	0.99 ± 0.79	0.88 ± 0.67	0.12	1.12 ± 1.06	0.93 ± 0.81	0.08
Glucose	134.75 ± 58.10	112.31 ± 53.37	0.001	136.84 ± 65.0	117.29 ± 54.56	0.01
Sodium	139.89 ± 3.23	140.09 ± 3.29	0.57	139.44 ± 3.4	140.05 ± 3.76	0.21
Potassium	4.47 ± 0.41	4.45 ± 0.42	0.59	4.45 ± 0.49	4.42 ± 0.47	0.65
Magnesium	1.89 ± 0.25	2.04 ± 0.25	0.02	1.86 ± 0.25	2.03 ± 0.29	0.01
Calcium	9.53 ± 0.68	9.45 ± 0.53	0.35	9.44 ± 0.68	9.37 ± 0.63	0.49
GOT	22.89 ± 10.08	23.59 ± 17.37	0.69	23.68 ± 14.08	29.97 ± 97.1	0.63
GPT	20.83 ± 13.88	22.83 ± 20.46	0.34	23.29 ± 21.66	24.35 ± 50.13	0.87

Abbreviations: GOT, glutamic oxaloacetic transaminase; GPT, glutamic pyruvic transaminase; HB, hemoglobin; PLT, platelets; WBC, white blood cells. * Missing data were observed for some parameters at specific time points, with up to 30% of the evaluated values unavailable.

**Table 3:** Major outcomes—assessment according to treatment group.

Characteristic	Study Group n = 137	Control Group n = 1787	*p*-Value
Suicide attempts	0 (0)	16 (0.9)	0.533
Medication dosage increases: 1–3 times	33 (24)	511 (28.5)	0.011
Medication dosage increases: more than 3 times	7 (5.0)	215 (12.0)	0.011
Change and/or addition of another psychiatric medication: 1–2 times	36 (26.2)	688 (38.5)	<0.001
Change and/or addition of another psychiatric medication: more than 2 times	1 (0.7)	90 (5.0)	<0.001
Amount of medication-associated negative events, level 1 *	45 (32.8)	690 (38.6)	<0.001
Amount of medication-associated negative events, level 2 **	16 (11.6)	407 (22.7)	<0.001

Data are presented as absolute numbers with % in brackets. * Level 1—At least one negative event in only one of the medication categories: a medication dosage increase, or a change and/or addition of another medication. ** Level 2—One negative event in the two different medication categories: a medication dosage increase, and a change and/or addition of another medication.

**Table 4:** Major outcomes—subgroup analysis according to psychiatric diagnosis.

Characteristic	BD ^§^	Schizophrenia ^§^	SAD ^§^	*p*-Value
Suicide attempts *				
Study group	0 (0)	0 (0)	0 (0)	---
Control group	3 (1)	9 (0.8)	4 (0.9)	0.94
Medication dosage increase *				
Study group	11 (29.7)	20 (26.6)	9 (36)	0.23
Control group	125 (43.4)	414 (38.5)	187 (43.8)	0.2
Change and/or addition of another medication *				
Study group	13 (35.1)	16 (21.3)	8 (32)	0.39
Control group	105 (36.4)	471 (43.8)	202 (47.4)	0.04

* Data are presented as absolute numbers with % in brackets. Abbreviations: BD, bipolar disorder; SAD, schizoaffective disorder. ^§^ The number of patients in each diagnosis, in the Study group and Control group, respectively, were as follows: BD—37 and 288; schizophrenia—75 and 1073; SAD—25 and 426.

**Table 5:** Major outcomes—age stratification.

Age Group Study Group ^§^ Control Group ^§^ Outcome *		≤40 Years n = 4 n = 699	41–65 Years n = 72 n = 709	≥66 Years n = 55 n = 305	*p*-Value ^¶^
Suicide attempts	Study group	0 (0)	0 (0)	0 (0)	---
	Control group	8 (1.1)	5 (0.7)	0 (0)	0.15
Medication dosage increases	Study group	1 (25.0)	25 (34.7)	14 (25.5)	0.51
	Control group	294 (42.1)	287 (40.5)	122 (40.0)	0.76
Change and/or addition of another psychiatric medication	Study group	2 (50.0)	23 (31.9)	12 (21.8)	0.28
	Control group	333 (47.6)	321 (45.3)	90 (29.5)	<0.001
Amount of medication-associated negative events	Study group	2 (50.0)	36 (50.0)	23 (41.8)	0.65
	Control group	452 (64.7)	447 (63.0)	153 (50.2)	<0.001

* Data are presented as absolute numbers with % in brackets. ^§^ The analysis included 131 patients in the Study group and 1713 patients in the Control group; missing data were observed for some parameters in the specific age groups—data were missing for 6 out of 137 patients in the Study group (~4.4%) and for 74 out of 1787 patients in the Control group (~4.1%). ^¶^ The *p*-values are given for within-group comparisons, i.e., comparisons between the different age-groups of the same treatment category (Study vs. Control).

**Table 6:** Multivariable logistic regression—predictors for the sum of medication-associated negative events.

Characteristics	OR	*p*-Value	95% Confidence Interval	
			Lower Limit	Upper Limit
Gender, male	0.983	0.86	0.808	1.195
Age, years	0.987	0.001	0.981	0.993
Ethnicity—Jewish (reference group)	1	----	----	----
Ethnicity—Arab	1.054	0.765	0.746	1.489
Ethnicity—Other	1.302	0.395	0.709	3.392
Sociodemographic status—high (reference group)	1	----	----	----
Sociodemographic status—medium	0.686	0.062	0.462	1.019
Sociodemographic status—low	0.682	0.035	0.478	0.973
Cerebrovascular disease	1.207	0.259	0.87	1.675
Myocardial infarction	1.016	0.954	0.601	1.717
Schizoaffective disorder	1.34	0.015	1.058	1.698
Bipolar disorder	0.662	0.009	0.486	0.902
Schizophrenia	0.746	0.015	0.589	0.945
No aspirin treatment (reference group)	1	----	----	----
Six-weeks to six-months of LDA treatment	0.426	0.004	0.24	0.759
Six-months to one-year of LDA treatment	0.96	0.896	0.521	1.769
One-year to two-years of LDA treatment	0.812	0.595	0.378	1.746

Abbreviations: BD, bipolar disorder; SAD, schizoaffective disorder.

## Data Availability

The original contributions presented in this study are included in the article/Supplementary Material. Further inquiries can be directed to the corresponding authors.

## Supplementary Materials

The following supporting information can be downloaded at https://www.mdpi.com/article/10.3390/ph19030435/s1: Table S1: Multivariable logistic regression—predictors for medication dosage increase; Table S2: Multivariable logistic regression—predictors for change and/or addition of another medication.
