# Supplementary material for: Does Administration of Low-Dose Aspirin Enhance the Efficacy of Psychotropic Drugs in Patients with Bipolar Disorder, Schizophrenia, and Schizoaffective Disorder?
Source: Pharmaceuticals (Basel). 2026 Mar 8;19(3):435. doi: 10.3390/ph19030435 (PMC13028740; doi:10.3390/ph19030435)
Supplement: Supplementary file 1 [file pharmaceuticals-19-00435-s001.zip › pharmaceuticals-4133046-supplementary.pdf]

Table S1. Multivariable logistic regression – predictors for medication dosage increase.

Characteristics	OR	*p*-value	95% Confidence Interval	
			Lower Limit	Upper Limit
Gender, male	1.079	0.446	0.888	1.311
Age, years	0.995	0.095	0.989	1.001
Ethnicity – Jewish (reference group)	1.0	----	----	----
Ethnicity - Arab	**1.435**	**0.037**	**1.022**	**2.014**
Ethnicity – Other	**2.149**	**0.01**	**1.201**	**3.486**
Sociodemographic status – high (reference group)	1.0	----	----	----
Sociodemographic status – medium	0.679	0.46	0.464	0.992
Sociodemographic status – low	0.774	0.135	0.553	1.083
Cerebrovascular disease	1.314	0.101	0.948	1.820
Myocardial infraction	1.234	0.444	0.72	2.114
Schizoaffective disorder	1.104	0.519	0.816	1.494
Bipolar disorder	0.905	0.519	0,669	1.225
Schizophrenia	**0.778**	**0.032**	**0.619**	**0.978**
No aspirin treatment (reference group)	1.0	-----	----	----
Six-weeks to six-months of LDA treatment	**0.309**	**0.001**	**0.152**	**0.630**
Six-months to one-year of LDA treatment	0.812	0.515	0.433	1.521
One-year to two-years of LDA treatment	0.986	0.971	0.453	2.143

Abbreviations: LDA, low-dose aspirin; OR, odds ratio.

Table S2. Multivariable logistic regression – predictors for change and/or addition of another medication.

Characteristics	OR	*p*-value	95% Confidence Interval	
			Lower Limit	Upper Limit
Gender, male	0.898	0.276	0.739	1.09
Age, years	**0.986**	**0.001**	**0.98**	**0.992**
Ethnicity – Jewish (reference group)	1.0	----	----	----
Ethnicity - Arab	0.967	0.848	0.686	1.363
Ethnicity - Other	1.458	0.205	0.814	2.612
Sociodemographic status – high (reference group)	1.0	----	----	----
Sociodemographic status – medium	0.82	0.306	0.561	1.199
Sociodemographic status – low	0.808	0.216	0.576	1.133
Cerebrovascular disease	1.058	0.741	0.758	1.476
Myocardial infraction	0.709	0.245	0.397	1.266
Schizoaffective disorder	**1.551**	**0.005**	**1.14**	**2.11**
Bipolar disorder	**0.733**	**0.025**	**0.558**	**0.962**
Schizophrenia	**1.364**	**0.025**	**1.039**	**1.791**
No aspirin treatment (reference group)	1.0	----	----	----
Six-weeks to six-months of LDA treatment	0.197	0.664	0.357	1.236
Six-months to one-year of LDA treatment	0.725	0.89	0.464	1.705
One-year to two-years of LDA treatment	0.153	0.528	0.220	1.269

Abbreviations: LDA, low-dose aspirin; OR, odds ratio.
